# Integration of a Technology-Based Mental Health Screening Program Into Routine Practices of Primary Health Care Services in Peru (The Allillanchu Project): Development and Implementation

**DOI:** 10.2196/jmir.9208

**Published:** 2018-03-15

**Authors:** Francisco Diez-Canseco, Mauricio Toyama, Alessandra Ipince, Silvana Perez-Leon, Victoria Cavero, Ricardo Araya, J Jaime Miranda

**Affiliations:** ^1^ CRONICAS Center of Excellence in Chronic Diseases Universidad Peruana Cayetano Heredia Lima Peru; ^2^ Institute of Psychiatry, Psychology & Neuroscience King’s College London London United Kingdom; ^3^ School of Medicine Universidad Peruana Cayetano Heredia Lima Peru

**Keywords:** mental health, mHealth, SMS, textmessaging, screening, mobile health, health services research

## Abstract

**Background:**

Despite their high prevalence and significant burden, mental disorders such as depression remain largely underdiagnosed and undertreated.

**Objective:**

The aim of the *Allillanchu Project* was to design, develop, and test an intervention to promote early detection, opportune referral, and access to treatment of patients with mental disorders attending public primary health care (PHC) services in Lima, Peru.

**Methods:**

The project had a multiphase design: formative study, development of intervention components, and implementation. The intervention combined three strategies: training of PHC providers (PHCPs), task shifting the detection and referral of mental disorders, and a mobile health (mHealth) component comprising a screening app followed by motivational and reminder short message service (SMS) to identify at-risk patients. The intervention was implemented by 22 PHCPs from five health centers, working in antenatal care, tuberculosis, chronic diseases, and HIV or AIDS services.

**Results:**

Over a period of 9 weeks, from September 2015 to November 2015, 733 patients were screened by the 22 PHCPs during routine consultations, and 762 screening were completed in total. The chronic diseases (49.9%, 380/762) and antenatal care services (36.7%, 380/762) had the higher number of screenings. Time constraints and workload were the main barriers to implementing the screening, whereas the use of technology, training, and supervision of the PHCPs by the research team were identified as facilitators. Of the 733 patients, 21.7% (159/733) screened positively and were advised to seek specialized care. Out of the 159 patients with a positive screening result, 127 had a follow-up interview, 72.4% (92/127) reported seeking specialized care, and 55.1% (70/127) stated seeing a specialist. Both patients and PHCPs recognized the utility of the screening and identified some key challenges to its wider implementation.

**Conclusions:**

The use of a screening app supported by training and supervision is feasible and uncovers a high prevalence of unidentified psychological symptoms in primary care. To increase its sustainability and utility, this procedure can be incorporated into the routine practices of existing health care services, following tailoring to the resources and features of each service. The early detection of psychological symptoms by a PHCP within a regular consultation, followed by adequate advice and support, can lead to a significant percentage of patients accessing specialized care and reducing the treatment gap of mental disorders.

## Introduction

### The Comorbidity of Mental Disorders and Physical Conditions

Worldwide, mental health disorders are highly prevalent and disabling conditions [1]. In Peru, mental disorders affect 1 in 5 people and are the leading cause of disease burden [2,3]. Certain population subgroups experience a higher burden of mental disorders, such as primary health care (PHC) services users. For instance, in Peru, the prevalence of depression is as high as 50% among patients with tuberculosis [2], 40% during pregnancy [3-5], 68% among female patients living with HIV or AIDS [6], and up to 57.8% for patients with diabetes [7-9]. In addition to this high comorbidity, for people living with a physical disease, depression has been associated with reduced treatment adherence, poorer prognosis, greater disability, and higher mortality [10-12]. Among pregnant women, depression is associated with underutilization of antenatal care services, premature birth, lower birth weight, and constitutes the main risk factor for postpartum depression [13-16]. Despite the high prevalence and negative impact on patients’ lives, depression and other mental disorders go largely underdiagnosed and undertreated [2,6]. Indeed, up to 85% of people reporting a need for mental health care declared not receiving any care [17,18].

### Strategies to Address the Comorbidity

Addressing comorbid physical and mental conditions, particularly within primary care, provides an opportunity to reduce the existing mental health treatment gap [19]. Accordingly, the World Health Organization proposes the integration of mental health services with general health care as a key solution to this gap, even in low- and middle-income countries (LMICs) [20]. Furthermore, there is growing evidence of the effectiveness of task shifting in the management of some chronic conditions, including mental disorders [21].

Mobile communications have also shown increasing potential for the improvement of health care [22]. Some mobile health (mHealth) strategies have proven to be useful in improving the efficiency of health care delivery, including interventions provided via technological platforms for the management of physical conditions [23-25], mental disorders [26-28], and as clinical decision support tools [29-32]. Moreover, strategies using short message service (SMS) as periodic reminders and informative messages have had a positive impact on medication adherence, appointment attendance, symptom monitoring, satisfaction with health services, and promotion of healthy behavior [27,33,34].

Packaging and deploying pragmatic strategies to introduce mental health screening and treatment within existing PHC platforms are needed in Peru and similar LMIC settings [35]. In this paper, we describe the design, development, and implementation of the *Allillanchu Project* and discuss the feasibility and challenges to its implementation at a larger scale.

## Methods

### Study Description

The *Allillanchu Project* aimed to design, develop, and test an intervention to promote early detection, opportune referral, and access to treatment of patients with common mental disorders attending public PHC services in Lima, Peru. *Allillanchu*, meaning *How are you? How are you feeling?* in *Quechua*, a Peruvian indigenous language, was chosen to emphasize the need to integrate mental health care into PHC providers’ (PHCPs’) routine practices. The intervention combines three key strategies: (1) training of PHCPs in the use of a screening tool to detect and manage depression, anxiety, psychosis, convulsive disorder, and alcoholism; (2) task shifting of detection and referral of mental disorders to PHCPs; and (3) an mHealth component that comprises a screening app plus motivational and reminder SMS text messages to patients.

### Study Design

**Figure 1:**
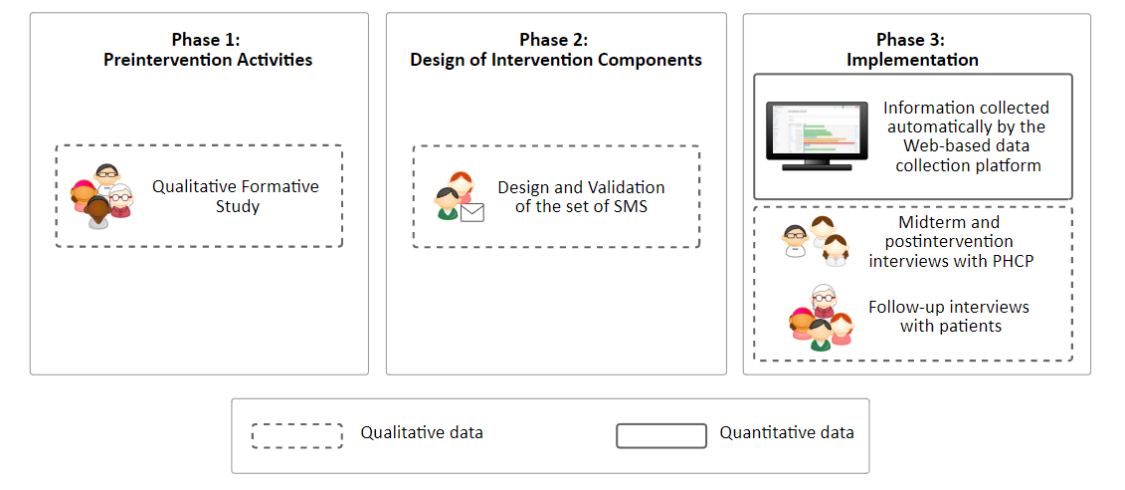
Collection of data in the study phases. SMS: short message service; PHCP: primary health care provider.

The project comprised three phases: phase 1, preintervention activities; phase 2, design of intervention components; and phase 3, implementation (see *Procedures*). In each of these phases, we collected quantitative and qualitative information. This project followed a mixed-methods approach, through a multiphase design [36]. This methodology is commonly used with feasibility studies. This design included both sequential and concurrent data collection. During phases 1 and 2, data were collected sequentially; and in phase 3, qualitative and quantitative data were concurrently collected (see Figure 1).

### Setting

In Peru, there are two main public health systems: the Ministry of Health and the social security system (EsSalud). The intervention was deployed in five different public PHC centers: three from the Ministry of Health and two from EsSalud. All of them served low-income populations living in the northern districts of Lima, Peru’s capital. The study involved PHCPs and patients from these five health centers that included at least one of the following health services (see Table 1): antenatal control (3 services), tuberculosis (4 services), chronic diseases (2 services), and HIV or AIDS (1 service). Each health service had between one and four PHCPs, each attending 15 to 20 patients per day, in 6-hour working shifts.

All participating health centers had at least one psychologist as staff; the only specialized mental health professionals available at the primary care level. Psychiatry specialists work in general and psychiatric hospitals; and patients had to be referred from PHC to be able to access these professionals.

Furthermore, the health centers from the social security system have a service of complementary medicine (ie, Tai chi) for referred patients to improve their physical health condition or their emotional well-being.

**Table 1:** Number of health centers and services included in the study.

Services	Ministry of Health			Social Security System (EsSalud)		Total
	Health center 1	Health center 2	Health center 3	Health center 4	Health center 5	
Tuberculosis	0	1	1	1	1	4
Antenatal care	1	1	0	1	0	3
Chronic diseases	0	0	0	1	1	2
HIV or AIDS	0	0	1	0	0	1
Total	1	2	2	3	2	10

### Participants

#### Primary Health Care Providers

A total of 22 PHCPs (12 midwives, 8 nurses, and 2 nurse assistants) out of 29 working in these facilities (76%) agreed to include the screening and the referral of positive cases as part of their routines during the 9-week study period.

#### Patients

Adult patients, aged ≥18 years and attending participating services, were invited into the study and had to consent to participate in the study. Participants who screened positive were recommended by the PHCPs to seek mental health care, received a set of tailored SMS text messages to motivate them to seek such care, and were contacted by the research team for a follow-up assessment (see *Phase 3: Implementation*).

### Procedures

**Figure 2:**
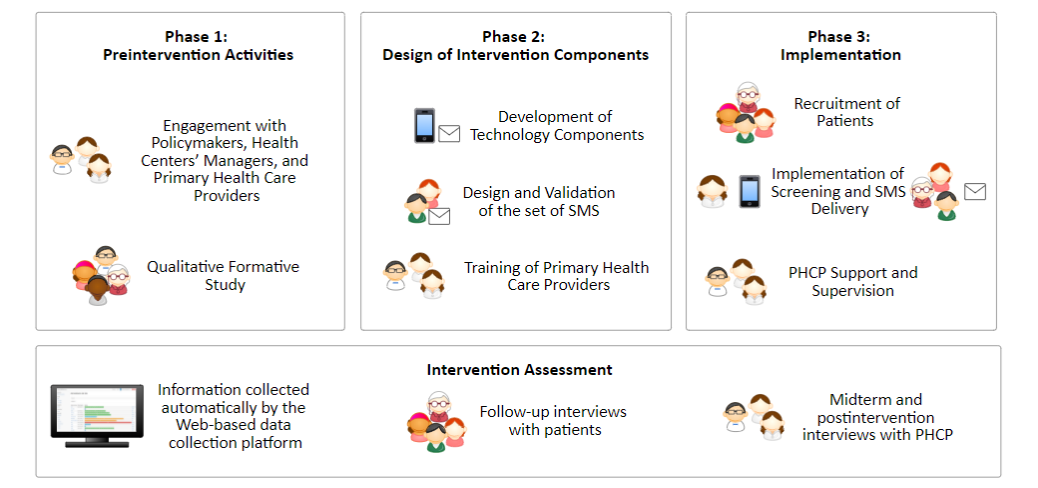
Allillanchu project’s phases. SMS: short message service; PHCP: primary health care provider.

The *Allillanchu Project* involved three different phases (see Figure 2):

1. Preintervention activities: explored the implementation setting to develop a suitable and context-specific intervention
2. Design of intervention components: developed the key components needed to deliver the intervention, including the training and supervision of PHCPs
3. Implementation: tested the integration of the mental health screening and referral in primary care combining the mHealth strategies, task shifting, and training of PHCPs.

### Phase 1: Preintervention Activities

#### Engagement of Policy Makers, Health Centers’ Managers, and Primary Health Care Providers

This stage consisted of a year-long period of meetings with policy makers and the personnel at the health centers, including the centers’ directors (n=5), heads of the services (n=10), and PHCPs (n=29). These meetings aimed to present the study, assess its feasibility, and secure the buy-in of managers to obtain the necessary approvals to implement the study in their facilities.

#### Qualitative Formative Study

From September 2014 to December 2014, we conducted a qualitative study to identify implementation barriers and facilitators. The research team interviewed patients and personnel of health centers where the intervention will later be implemented, including 22 PHCPs, four clinical psychologists and 37 patients. The interviews were conducted using semistructured guides, and the duration was approximately of 45 min. All interviews were recorded, transcribed verbatim, and analyzed by the research team using Atlas.Ti, version 7 (Scientific Software Development GmbH).

The main results of this qualitative formative study were as follows: (1) screening for mental disorders was not a quotidian practice in the services, (2) PHCPs were willing to introduce the screening in their routines but anticipated time constraints, (3) though it was unusual for them to do so, many patients were willing to seek mental health care if they were advised to do so, (4) mental health specialists were scarce in participating health centers, and (5) interviewed clinical psychologists recognized the value of introducing a mental disorders’ screening in primary care but were concerned about PHCP’s ability and willingness to do so. Findings of the qualitative study will be reported in detail elsewhere.

### Phase 2: Design of Intervention Components

We proposed the inclusion of a technology-based screening tool into the routines of trained PHCPs, complemented by a set of automated SMS text messages to encourage patients who screen positive to seek specialized care and to advise on where to seek help.

#### Development of Technology Components

The technology consisted of three integrated components: a screening app, a Web-based data collection platform, and an automated SMS text message delivery app.

The *screening app*, installed on a tablet, used the Self Report Questionnaire (SRQ). The guiding principle was to develop an app to (1) Produce immediate results on symptomatology that may require professional help, (2) Offer guidelines to PHCPs to advise the patient on how and where to seek help, and (3) Ask PHCPs to report on actions taken with positive cases (ie, recommendation to attend to a psychology service). The SRQ [37], a screening tool developed by the World Health Organization, is recommended in the current mental health reform in Peru as the tool to be used in PHC. The SRQ version used by the Peruvian Ministry of Health consists of 28 yes or no questions, 18 of which screen for depression and anxiety, four for psychosis, one for convulsive disorder, and five questions for alcoholism (see Multimedia Appendix 1). The Peruvian social security system was planning to implement a shorter version of the SRQ in their services at the time; therefore, these centers used a short version, which includes the first 18 items assessing depression and anxiety. The team further added two questions to assess suicide risk. These questions were asked only when patients answered positively to question 18 of the SRQ, which establishes the presence of suicidal ideation. Each PHCP had a personal log-in that displayed the SRQ version for their health system, Ministry of Health, or EsSalud.

We collected user feedback on the screening app through a small pilot with five PHCPs from different health services (n=4) and centers (n=3) for 2 weeks. The pilot found that PHCPs had no difficulties using the screening app; they found it easy to use and were able to include it in their daily routines. In the 2-week pilot, PHCPs screened a total of 47 patients.

**Figure 3:**
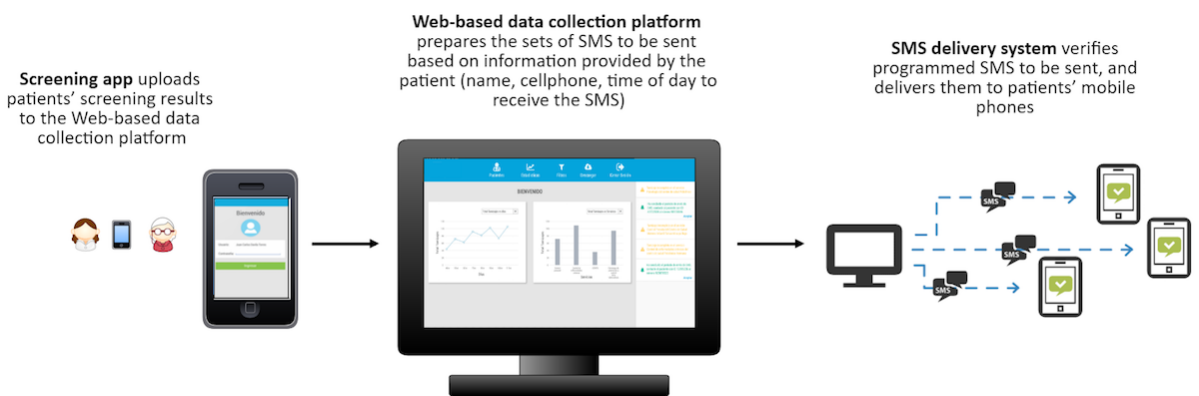
Integration of technology components. SMS: short message service.

The *Web-based data collection platform* stored all the information collected by PHCPs using the screening app, allowing the research team to monitor progress in real time (see *Intervention Assessment*). As patients were screened, the app uploaded the results to the Web-based data collection platform in real time using a mobile data connection. Furthermore, the information provided by the patient during the recruitment process was also uploaded to the Web platform (name, cellphone number, and time of day to receive the SMS text message) and consolidated under a patient ID. Thus, based on the screening results, the Web platform was able to match a positive screening under the specified ID with the patient’s personal data to automatically send an SMS text message (see Figure 3).

#### Design and Validation of the Set of Short Message Service

The research team developed a set of five SMS text messages to further motivate patients to seek mental health care. Patients who screened positive and were referred to specialized care received three SMS text messages per week, during 2 weeks. Of the five SMS text messages, sent every other day, one aimed to remind patients where and when to find mental health care (*reminder SMS text message*), whereas the other four sought to motivate patients to seek help (*motivational SMS text message*) addressing either barriers or facilitators for accessing mental health care. The pattern of SMS text message delivery over the 2 weeks was the same: day 1: reminder SMS text message, day 2: motivational SMS text message, and day 3: motivational SMS text message. As the reminder SMS text message was sent twice, each patient received a total of six SMS text messages over the 2-week period.

All SMS text messages were designed and validated through two pilot studies, conducted from December 2014 to January 2015, with 63 patients with similar characteristics to those involved in the implementation phase. The first pilot study aimed to explore (1) Patients’ use of mobile phones and SMS text messages and (2) Barriers and facilitators for seeking mental health care. On the basis of these results, we developed seven SMS text messages that were validated and ranked by patients in a second study [38]. This resulted in the selection of the set of five SMS text messages for the implementation phase that (1) Were simple and direct, (2) Avoided mentioning health conditions (eg, depression), (3) Were tailored with the patient’s name and health centers’ name as signature, and (4) Addressed the barriers (ie, lack of time and money) and highlighted the positive effects of looking for specialized mental health care (ie, feeling better, having someone to talk to, and receive guidance), as reported by the interviewed patients.

#### Training of Primary Health Care Providers

To participate in the implementation, the 22 PHCPs were trained in the use of the screening app. This training consisted of two sessions of 8.5 hours each, which were developed and offered by the project team, which included psychologists, one sociologist, and a consultant with a background in psychotherapy. This activity sought to offer knowledge, skills, and motivation to PHCPs to enable the routine screening and referral of patients to available mental health services as indicated by the app. The mental health content was based on the mhGAP training modules [39]. It included presentations, role-playing, and practical exercises.

### Phase 3: Implementation

#### Recruitment of Patients

From September 2015 to November of 2015, a team of 10 recruiters enrolled patients (see *Description of Study Participants*). The recruitment took place in the waiting rooms before the patients’ appointments and involved them providing informed consent to be part of the study and providing personal data to be uploaded to the Web-based data collection system. The informed consent explained that in the case of a positive screening during one of their regular consultations, they would receive SMS text messages and have a follow-up interview with the research team.

#### Screening Implementation and Short Message Service Delivery

**Figure 4:**
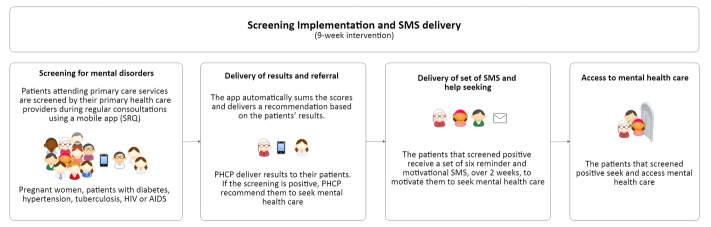
Intervention procedures. SRQ: Self-Report Questionnaire; PHCP: primary health care provider.

The 22 trained PHCPs were invited to use the screening app in their regular consultations during 9 weeks, from 2015 September to November of 2015, and to refer patients with a positive result to mental health care—either psychology service, complementary medicine, or general medicine—according to each center’s protocol. The tablets were provided by the *Allillanchu Project* and included a mobile data plan to upload the information to the Web-based data collection platform in real time (see Figure 4).

#### Primary Health Care Provider’s Supervision and Support

Over the 9 weeks, the research team provided telephone and face-to-face support and supervision to the PHCPs to ensure the correct implementation of the screening. The Web-based data collection platform was monitored on a daily basis to identify PHCPs who were not using the app as intended and to contact them to assess and solve the situation.

All of the PHCPs had supervision meetings with the research team at least twice a week. In these meetings, support was offered by solving any issues or doubts regarding the use of the tablet or the app and by accompanying the PHCPs in managing potentially difficult situations. Problems with the administration of the SRQ were also assessed and solved. PHCPs could initiate contact with the research team through a “help” function installed in the app or a phone call.

### Intervention Assessment

To assess if and how the intervention promoted early detection, opportune referral, and access to treatment of patients with mental disorders, we used the following methods and sources of information:

1. *Information collected automatically by the Web-based data collection platform.* The platform collected and provided information on the screening progress by health center, service, PHCP, patient, and over time, including the number of screenings (completed and incomplete), number of positive cases by type of disorder and of suicide risk, and actions taken by the health provider with each patient (eg, referral to the psychologist). It also included demographic data of all the screened patients.
2. *Follow-up interviews with patients* (Multimedia Appendix 2) *.* We conducted face-to-face structured postintervention interviews with patients. The interview explored if the patient had sought and received mental health care after being referred by the PHCP, their motivation to seek—or not—specialized care and how the SMS text message encouraged them—or not—to do so, as well as perceived barriers and their opinion on received care. The research team interviewed patients 3 weeks after they screened positive.
3. *Midterm and postintervention interviews with PHCPs* (Multimedia Appendix 3) *.* Face-to-face semistructured interviews were conducted halfway through and at the end of the intervention. The midterm evaluation assessed PHCPs’ personal experiences with the screening (eg, use of technology and delivering results to patients) and aimed to identify and solve problems. The postintervention evaluation explored the experiences and opinions of participating PHCPs, to identify barriers and receive suggestions for improvement, and to assess the perceived feasibility and willingness to continue implementing the screening.

### Data Analysis

The screening and referral of patients’ data, stored in the Web-based data collection platform, was exported and analyzed using Statistical Package for the Social Sciences (SPSS) statistics for Windows, version 23.0 (IBM Corp) through descriptive analyses, by reporting frequencies and percentages.

Information from patients’ and PHCP’s interviews was registered and analyzed in a similar way. All interviews were audiorecorded, transcribed, and summarized in a matrix. The qualitative information was organized by participant (rows) and by themes of each interview guide (columns) [40] (Multimedia Appendix 4). Each theme included a summary-cell of interviewee experiences or opinions, as well as quotations. Data were organized and coded by three members of the research team after an initial period of standardization of criteria and practice. The answers to closed-ended questions (eg, “Did you seek care from a general practitioner or psychologist as recommended?”) were analyzed, reporting frequencies and percentages.

### Ethics

The study protocol, informed consent forms, and instruments were approved by the institutional review board of the Universidad Peruana Cayetano Heredia. The study was also approved by the directors of the five participating PHC centers. Participant PHCPs signed a consent form that described the project goals and procedures, as well as their commitment, the benefits and incentives of participation (which comprised a training-assistance certificate), and funding support toward a one credit brief course of their choice, for up to US $30, at the end of the study.

Patients provided oral informed consent on the following components, which would be implemented only in the case of positive screening results during one of their regular consultations: (1) receive six SMS text messages over 2 weeks, (2) have a follow-up interview, and (3) consent for the interview to be recorded. Ethical approval was also obtained for collection of personal data (name and cellphone number) during the design and validation of the set of SMS text messages.

## Results

### Description of Study Participants

A total of 22 PHCPs (12 midwives, 8 nurses, and 2 nurse assistants), all female, participated in the study. During recruitment, 2580 patients (22.4% men) attending the selected facilities were invited to participate in the study, and 1772 (68.7%) accepted (see Table 2). The average age of enrolled participants was 42.8 years (SD 20.3).

### Implementation of the Screening

The PHCPs performed 762 screenings with 733 patients, as 29 of these were screened for a second time in a following consultation. During the intervention period, the average number of screenings per week was 85 (SD 38). However, the number of screenings per week varied and tended to progressively decrease, having its highest and lowest points during the second (159 screenings) and last week of the intervention (52 screenings), respectively.

Chronic disease services had the highest number of screenings, accounting for almost half of the 762 screenings (49.9%, 380/762), followed by the antenatal care services (36.7%, 280/762), the tuberculosis services (12.1%, 92/762) and the HIV or AIDS services (1.3%, 10/762), respectively.

### Positive Cases Detection and Referrals

Out of the 733 patients screened, 159 of them (21.7%) had a positive result by the SRQ. Of those 159, 150 (94.3%) screened positive for one disorder and 9 (5.7%) for two disorders.

Distributed by health service, the highest prevalence was found in the HIV or AIDS service according to the SRQ, where 7 out of 10 screened patients were positive (70%), followed by the chronic diseases services (90/364, 24.7%), antenatal care services (49/274, 17.9%), and tuberculosis services (13/85, 15.3%).

The most prevalent mental disorders were depression or anxiety, grouped as a single condition in the SRQ, with 125/733 patients screening positive for at least one of these conditions (17.1%). At the Ministry of Health centers, who implemented the 28-item version of the SRQ, 41/194 screened patients (21.1%) answered positively to at least one of the four items for psychosis, and 2 (1%) were positive for alcoholism. No cases of convulsion were detected. Additionally, 22 of all 733 screened patients (3%) reported a current suicidal ideation.

According to the PHCPs’ reports, the vast majority of the 159 cases detected were referred to mental health care. These referrals consisted in advising their patients to seek specialized care at their health center. Of these 159 patients, 107 (67.3%) were referred to the psychology service; 27 (17%) of the cases were accompanied by the PHCP to a health services (psychology, general medicine) to facilitate access; 4 (2.5%) of the suggestions involved seeking care at a different service (complementary medicine, general medicine); 11 (6.9%) were patients already receiving specialized care and were advised to continue their treatment; 6 (3.8%) reported other actions, that is, providing a piece of paper with the health service of referral written and the signature of the PHCP; and 4 (2.5%) omitted reporting the actions taken.

### Help-Seeking and Access to Mental Health Care

**Figure 5:**
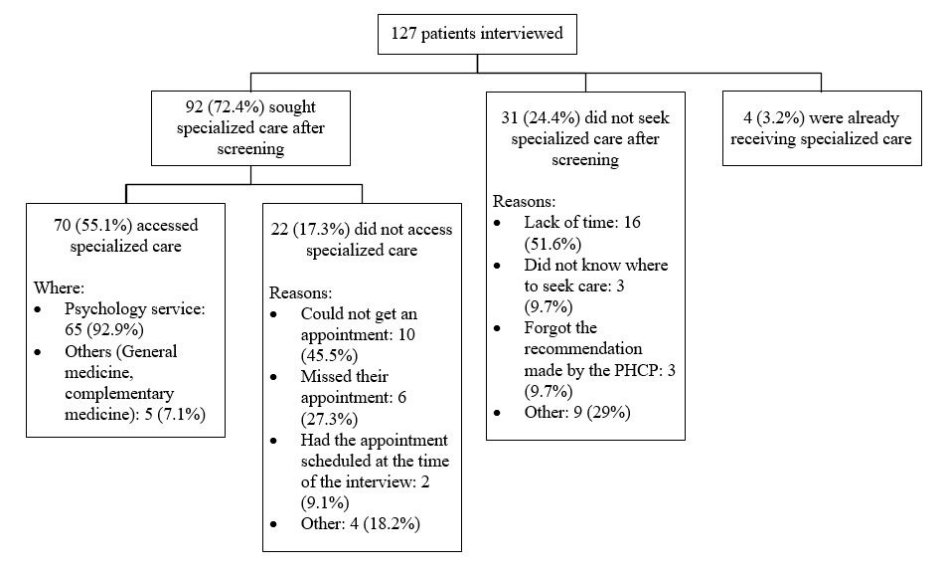
Breakdown of patients interviewed.

Out of the 159 patients with a positive screening result, 143 previously gave their consent to participate in the follow-up interview, and 127/143 patients (88.8%) were interviewed. The average age of interviewed participants was 49 years (SD 19), 101/127 (79.5%) were women, and 92/127 (72.4%) reported having sought specialized care after receiving the advice of the PHCP. In addition, 70/127 (55.1%) accessed care, having had at least one consultation in a specialized service after the screening (see Figure 5).

**Table 2:** Characteristics of enrolled patients.

Characteristics		n (%)
**Sex**		
	Male	346 (19.5)
	Female	1426 (80.5)
**Health system**		
	Ministry of Health	609 (34.4)
	Social security system (EsSalud)	1163 (65.6)
**Health service**		
	Antenatal care service	931 (52.5)
	Chronic diseases service	709 (40)
	Tuberculosis service	121 (6.8)
	HIV or AIDS service	11 (0.6)

All patients who screened positive received a total of six SMS text messages over 2 weeks. However, only 35/127 interviewees (28%) were eligible to answer questions about the perceived effects of the SMS text message, as many participants had already sought specialized care before receiving the first SMS text message, therefore defeating their motivational purpose. Others did not seek specialized care, did not consent to receive the SMS text message, or reported having problems with their cellphone. Of the 35 patients who were suitable to discuss the perceived effects of receiving the SMS text messages, 30/35 (85.7%) considered the set of SMS text messages to be effective in promoting motivation to seek care.

During the interviews, the patients tended to focus on the difficulties and symptoms they were facing, but also offered points of view that further support the implementation of the screening and showcase the benefits of accessing a mental health specialist, as illustrated in the following quotes:

I think it is very good that midwives ask their patients how they feel [...] for example, when I came I was going through a deep depression and I wasn’t accepting my pregnancy. After the midwife asked me some questions, she sent me to the psychologist of the health center and he instantly saw me. We had a conversation of at least an hour and a half, he helped me a lot. Thanks to them, my depression has decreased, now I feel better, calmer.

Patient of antenatal care, female, 29 years

The psychologist treated me really well, he made me notice some things and told me it was necessary that my wife come to see him as well, because most of the problems I had were due to our conflictive relationship. Now I feel much better, I am trying to do my best, I am trying to stop having negative thoughts, of eliminating me [...] I want to have another appointment with the psychologist, because with the help of a professional I can receive guidance to think in a different way.

Patient of the chronic diseases service, male, 64 years

### Primary Health Care Provider’s Assessment of the Project

Of the 22 PHCPs, 21 were interviewed for the midterm evaluation and 22 for the final evaluation.

#### Training

As part of the training, a pre- and posttest evaluation was conducted with a maximum possible score of 20 on each test. The average score at pretest was 12.1 (SD 2.3) and 16.5 (SD 1.35) at posttest, showing an increase of 4.4 points toward the end of training.

In addition, the participating PHCPs assessed the training at the end of the sessions and after the 9-week intervention. They believed the training made them more aware of the importance of caring for people’s mental health, provided them with knowledge and skills in mental health—an understudied topic in their professional education—taught them how to use a screening tool to assess their patients’ mental health, and offered them necessary skills to help their patients. However, almost half of the PHCPs suggested more time to be allocated to discussing the questions of the SRQ, the screening tool.

#### Screening

When asking to assess their ability to implement the task as intended, the answers showed a great variability among the PHCPs of the different health services involved. Specifically, most of the midwives from the antenatal care services reported not being able to implement the screening as part of their routine because of the high workload and the great amount of paperwork for their current tasks, as illustrated in the following quote:

Some days I was screening and I was really excited to do it, but when I was with the third pregnant woman screened, I looked at my watch and it was already 10:30 am, and by noon I had to see 12 women. That meant that I had to stop using the tablet and rush to finish on time with all 12 consultations from my shift.

Midwife, antenatal care service

Nurses from the chronic disease service reported being unable to do the screening when conducting the weekly educational workshop on disease management with their patients. Conversely, the tuberculosis service’s nurses were able to schedule the screening with their patients. One nurse assistant stated the following:

We had a list of patients and they had already agreed to answer the screening so we would schedule to do the screening on a specific day when they come to take their medication for tuberculosis.

Nurse assistant, tuberculosis service

In addition, as the intervention was tested during PHCP’s routine activities, they were unable to implement the screening in all of their shifts because of other responsibilities, rotation to other services, holidays, and community activities. Furthermore, the way the screening task was organized was highly variable between and within the health services. Indeed, there was a high variability regarding the moment in which the screening was performed throughout the consultation, even among PHCPs within the same service. Although some reported doing it at the beginning, others included it at the end of the consultation. Despite this variability, most of them agreed that they would most likely do the screening if their workload was lower or if they had a colleague to relieve the workload during the consultation.

The main challenge was related to specific questions of the screening test. The questions about psychosis were difficult for the PHCPs to score because some patients did not understand the questions about delusional beliefs (see Multimedia Appendix 1, items 19 and 20). The questions about physical symptoms were challenging because of the difficulty of discerning if the symptoms were caused by their physical condition or an underlying mental problem. However, the PHCPs were able to overcome these challenges by using strategies learned in the training sessions, such as explaining the questions to their patients, paraphrasing, and adding a follow-up question for clarity.

Regarding technology, despite PHCPs having different knowledge of tablet-use, no difficulty of use was reported. Additionally, they noted that, compared with a printed version, the app facilitates the task, as they are overburdened with paperwork and believe a new paper-form would add to their current burden.

Overall, when asked about the screening task, the PHCPs gave very positive feedback. Most considered that they were able to provide a more comprehensive care by addressing problems that they may have already identified but did not know how to handle. In addition to this, they mentioned being able to bond with their patients through listening and learning more about them. Some PHCPs also reported being more aware of the importance of their patients’ mental health because of its effect on their physical health and personal lives. Furthermore, some of the PHCPs declared that even after ending the intervention, they continued to ask their patients about their emotional well-being. One nurse stated the following:

We have been able to improve our consultations and to make patients feel that we want to do more, that is not only a check-up, give them pills and that is all. We have let them know that we care and we can give them more, and it is our intention to do so, even though sometimes we have limitations.

Nurse, chronic disease service

#### Delivery of Results and Referral

Messages conveyed as part of the delivery of the screening results varied widely among PHCPs; nonetheless most of them revolved around the importance of seeking specialized care.

Of the 22 PHCPs, 17 (81%) reported no difficulties with the delivery of screening results. However, some reported difficulties regarding their ability to address mental health issues. Some of the PHCPs found the suicide risk cases particularly challenging and felt unsure about how to handle them. Other less frequent challenges were feeling emotionally affected by the patients’ stories and not feeling prepared to offer support, as illustrated in the following quote:

For me it was a little difficult to manage the patient that had suicide risk. It was a surprise to know that a patient had that problem, because apparently, she was a strong woman [...]. The patient had this problem during all the pregnancy, and I did not realize it until the screening and that made me feel bad. Maybe more patients come with the same condition and one does not realize it.

Midwife, antenatal care service

The PHCPs stated no difficulties with the referral process, with most of the patients being open to seeking specialized care. The PHCPs reported being able to report the actions taken after a positive screening through the app. Furthermore, some PHCPs reported referring patients who, despite having negative screening result, showed symptoms that they deemed relevant.

The referral procedure was different across services. The most common ways were personally escorting the patient to the psychology service, arranging an appointment for the patient, and giving the patient a referral paper for them to seek an appointment with the psychologist. In the services with less patients, such as the tuberculosis and HIV or AIDS, it was more common for the PHCPs to escort their patients to the psychologist’s office, whereas it was more common to offer written referral papers in antenatal care and chronic disease services, where only severe cases such as suicide risk were escorted as required by the study protocol.

Though PHCPs were also able to refer their patients to a general practitioner, most of them preferred not to do so, with only five PHCPs reporting having referred a patient to a general practitioner. This was because of the perception that the general practitioner was more suited to treat physical conditions, whereas the psychologist was more appropriate to treat mental health.

#### Feasibility of Integrating a Mental Health Screening

The majority of PHCPs reported willingness to continue implementing the screening in their services. However, they also noted that time constraints would be a key barrier to do so on a regular basis. Moreover, screening activities could not be reported in the medical records, which meant that these would not count toward their productivity assessments. Most of the PHCPs agreed that they would most likely do the screening when less burdened by workload or if they had a colleague to offer support during their working hours. One nurse stated the following:

We are pressured to complete four consultations per hour, and you may think that is not much, but attending four patients take a lot of your time. If the screening is implemented permanently, I think we would have to reduce the number of consultations per hour to three, that way we would have more time. Another solution would be that of our six-hour shift, we only have patients scheduled for five hours, and the spare hour could be used to extend the consultation time for the patients, if needed. There are a lot of possible solutions; it is only a matter of disposition to find them.

Nurse, chronic diseases service

Another barrier is the perception by PHCPs that there are not enough psychologists to attend all patients requiring help and that services hours are limited—those who are available may not be present in their services all day because they conduct activities other than consultations and some services only operate in the mornings, thus limiting availability.

To tackle these barriers, PHCPs proposed 3 main suggestions to improve the feasibility and sustainability of the screening: (1) 8 PHCPs (36.4%, 8/22) suggested including the screening as formal procedure in their services with allocated time, thus improving the conditions to accommodate this new task without being overburdened, that is, raise the time limit for each consultation and lower the daily patient workload; (2) 8 PHCPs suggested changes or improvements to the screening test to simplify the questions and make them more comprehensible to the patients; and (3) 3 PHCPs (14.3%, 3/22) advised securing enough specialists to look after the referred patients.

## Discussion

### Principal Findings

The *Allillanchu Project* aimed to develop and test a multicomponent intervention to improve early detection, opportune referral, and access to treatment of patients with mental disorders attending public PHC services in low-income areas in Lima, Peru. With more than 750 screenings completed in real-world circumstances over a 9-week period, the *Allillanchu Project* showcases the feasibility to integrate the mental health screening into primary care services as a routine procedure. The use of brief screening tools is considered an important first step to integrate mental health care into existing PHC services [41] and is aligned with the proposal of task shifting some mental health care activities, which has been successfully tested in other LMIC settings [42].

With basic training, supervision, and an easy-to-use screening app, PHCPs could be both willing and able, during their regular consultations, to identify symptoms of mental disorders among their patients and refer them to specialized care. The training was key to sensitize PHCPs about their patients’ mental health and strengthen their abilities to perform the screening, which is reflected in the improvement of scores at pre- and posttest. Likewise, the supervision was important to provide support when needed, solving doubts with the screening, and the use of technology. Interestingly, the screening was not only effective in many services but also well received by patients, who accepted to be screened and, furthermore, the large majority sought mental health care following the recommendation implemented as part of the project. The results of the screening and health seeking are aligned with the available evidence, which affirms that short-term training with ongoing monitoring and supervision of nonspecialist health workers can improve “confidence, detection, treatment, as well as treatment adherence of individuals with mental disorders” [42]. A relevant component of the intervention was the screening app, which proved to be a successful tool to optimize the detection and referral processes, as well as to monitor its implementation. The benefits of technology to simplify and shorten procedures within the health system have been extensively reported [26-32].

However, it is also true that the inclusion of the screening into the PHCP’s routines had both qualitative and quantitative differences. Qualitatively, each service organized the implementation of the screening in different ways, which can be considered a positive sign of adaptation and flexibility to achieve the task [43,44]. Quantitatively, some health services screened many more patients than others. One reason for this is that some services, for example, tuberculosis and HIV or AIDS, have fewer patients than other services. The most significant factor to explain the differing performance among PHCPs was the limited allocated time per consultation combined with the overwhelming patient workload and paperwork burdening only some services, such as antenatal care, leading to saturation, which has been seen in PHC settings in developed countries [45]. These were the two main implementation barriers to regular screening and were especially critical in the antenatal care services. Typically, each service has an established time to complete a set of standard procedures and register them in the clinical records. Therefore, implementing a new task would involve doing more work within the same time period. This observation of a decline in the number of screening in those weeks may be related to the fact that introducing screenings is an activity that competes with many other tasks. Although the general trend suggests that the screening was indeed adopted as part of their routine practices, it also shows that such tasks were not constant over time, thus calling for ongoing monitoring of these efforts in the future.

The accuracy of the screening was challenged by the ambiguity of some items of the psychosis subsection of the SRQ, the 28-item version recommended by the Ministry of Health. Despite its evidenced ease of use, patients found some of the items difficult to understand and thus, complicated PHCP’s scoring when administering the test. This difficulty to understand the questions probably explains the unexpected number of patients testing *positive* for psychotic symptoms, by answering *yes* to any of the four psychosis questions (eg, *Do you feel you can do things that others cannot do, or that you are a particularly important person?*), which indicates a positive case. This highlights the importance of improving the cultural validation of screening tools [41], as well as the importance of continuous training to improve the abilities of PHCPs to formulate the questions and to assess whether the answer provided by the patients matches the symptom described in the question.

In this project, the implementation of the screening revealed that an important proportion of patients regularly attending PHC services to treat a physical condition concomitantly have psychological symptoms that are probably ignored during their consultations. This prevalence, combined with a regular attendance of patients and a closer patient-provider relationship, confirms that performing the screening in PHC is an opportunity for an early detection and opportune referral of patients in need of care [41] An example of the potential benefits of a good screening in primary care is provided by a review of 40 international studies that show that, on average, 3 of 4 suicide victims had contact with a PHCP within the year of suicide, and 45% had it the month before [46].

Existing guidelines recommend screening the general adult population for depression [47,48]. One main goal of our study, besides the introduction of a screening into PHCP’s routines, was to increase access to mental health treatment. Remarkably, the referral process had better results than expected. Out of 10 patients, 7 actively sought specialized care, and 5 obtained a consultation with a mental health specialist, thus showing promising results for this type of intervention. Our results contrast sharply with existing practices in Lima, where only 32.8% of those who need mental health treatment report accessing it [49], and with studies in African and European hospitals, where only between 2% and 20% of users with mental disorders actually accessed a specialized consultation [50-52]. Two elements explain this success. First, PHCPs provided opportune advice to positive cases and made a substantial effort to guarantee specialized consultations on the same day of the screening for some of their patients. Second, SMS text messages were highly valued by participants who received them before seeking specialized care, translating into an inexpensive way to reaffirm the advice given by the PHCP.

Yet, several health system barriers remain in place: insufficient information about mental health services and costs, limited time to have an appointment, and scarce availability of mental health specialists, among others, which align with local studies’ findings [53-58], as well as our own during the formative study.

### Implications for Public Health

One of the most significant strengths of the *Allillanchu Project* was its development and testing through a rigorous multiphase design, set within the current organization of the Peruvian health system, and aligned with the national mental health reform [59]. This is positive in terms of sustainability of the innovation, allowing us to propose context-specific solutions to real-world conditions.

Shifting the detection and referral of people suffering mental disorders to nonspecialized health providers seems to be the right path to alleviate an overburdened health system with scarce specialized resources. However, some conditions need to be improved to make it feasible and sustainable. First, the detection and referral have to be recognized by the public health system as part of the PHCP’s tasks, allocating and protecting time for this activity within regular consultations and including it in the patient’s clinical record. Second, to ensure the access of referred patients to appropriate treatment, key barriers need to be addressed: informing patients about the availability, time, and cost of services; expanding the hours of consultation; offering free essential mental health care; transferring evidence-based treatments to nonspecialized PHCPs; and increasing the number of mental health specialists. Some of these improvements are already set as goals of Peru’s mental health reform [35,59]. Third, it is essential to engage policy makers, health providers, mental health specialists, and users in the effort of integrating mental health care as part of the regular care, caring for potential concerns, and resistance to the innovations by each of these groups.

It is also important to adapt the detection and referral processes to the working dynamics of each service, which vary according to resources and workload. This adaptation may include, for example, for each service to define screening frequency or the prioritization of screening for a subgroup of patients at higher risk of mental disorders.

Training and supervision are both essential to sustain a task-shifting strategy. On the basis of the *Allillanchu Project* experience, training in mental disorders detection and management should be regularly provided to PHCPs, and it should focus on developing skills to manage high-risk patients, to guarantee a more accurate application of the screening tool, and to plan how to introduce the screening with each PHCP in his or her health service. Additionally, as some PHCPs reported being concerned about the impact their patients’ accounts had on them, it would be important to include self-care strategies in the training sessions. Considering the time constraints faced by PHCPs and the challenges with the SRQ’s psychosis items, it could be appropriate to consider using a shorter instrument focused on fewer disorders such as depression and anxiety because of their higher prevalence among the Peruvian population [49,54-57]. Additionally, exploring alternatives to tackle this issue in future studies, such as self-administered screening test for patients, may contribute to overcome these hurdles.

In terms of sustainability of the mHealth-based screening within the public health system, considering its potential to improve the health system’s efficiency, as well as its potential to centralize patients’ information and articulate it with other technology-based health initiatives, for example, electronic health records, it would be advisable to begin to use the mHealth-based screening at a larger scale.

### Study’s Strengths and Limitations

A major strength of our study was its design and implementation in real-world settings, through accommodating policy makers’ preferences, adapting to PHCPs and health services’ structures and organization, and creating links between patients who needed specialized care with available care. This strength was paired with the quasi-experimental study design of the implementation phase, which, through using a mixed-methods approach, revealed that no other external factors that could have simultaneously influenced the screening, referral, and access to treatment of participants at risk of developing mental disorders were in place.

Yet, some limitations are worth noticing. For example, the help-seeking responses and access to specialized mental health care, after screening, were self-reported by the patients during a follow-up interview. As we did not have access to the health system records, self-reported information was the best way to collect such information. Another limitation was that only a proportion of participants with a positive screening were eligible to report on perceived effects of the SMS text messages on their help-seeking behavior, largely because of many of them seeking and receiving care before the SMS text messages reached them. This unexpected “positive” result, though less beneficial to the assessment of the SMS text message component in terms of numbers of interviewees, reveals a very promising direction toward the efficacy of screening and referral within nonspecialized services.

### Conclusions

The use of a screening app by nonspecialized PHCPs, supported by basic training and supervision, is a feasible procedure and confirms a high prevalence of undiagnosed psychological symptoms among regular users of PHC services. However, for it to be made a routine, the health system needs to formally accommodate it as a PHCP’s task and remove its major barriers, particularly time constraints and availability of specialized mental health personnel. To increase their usefulness and sustainability, the detection and referral of cases should be tailored to the workload, resources, and organization of each service. The early detection of psychological symptoms within a regular consultation, followed by adequate advice and motivation, can lead to an important proportion of patients to seek and access specialized care, thus optimizing the use of existing resources and reducing the treatment gap of mental disorders.

## Appendix

Multimedia Appendix 1 Self-Reporting Questionnaire (SRQ).

Multimedia Appendix 2 Follow-up interview guide with patients.

Multimedia Appendix 3 Midterm and postintervention interview guide with PHCP (primary health care provider).

Multimedia Appendix 4 Analysis of patients' and PHCPs' (primary health care provider) interviews.

## Abbreviations

LMIC: low- and middle-income countries
mHealth: mobile health
PHC: primary health care
PHCP: primary health care provider
SMS: short message service
SRQ: Self Report Questionnaire
