# Supplementary material for: Integration of a Technology-Based Mental Health Screening Program Into Routine Practices of Primary Health Care Services in Peru (The Allillanchu Project): Development and Implementation
Source: J Med Internet Res. 2018 Mar 15;20(3):e100. doi: 10.2196/jmir.9208 (PMC5893885; doi:10.2196/jmir.9208)
Supplement: Multimedia Appendix 1 [file jmir_v20i3e100_app1.pdf]

Multimedia Appendix 1

Self-Reporting Questionnaire (SRQ)

Instructions:

I would like to ask you some questions about any bother or difficulty that you may have felt during the last month. Please, answer Yes or No to each one of the questions*

Questions	Yes	No
1. Do you often have headaches?	Yes	No
2. Is your appetite poor?	Yes	No
3. Do you sleep badly?	Yes	No
4. Are you easily frightened?	Yes	No
5. Do your hands shake?	Yes	No
6. Do you feel nervous, tense or worried?	Yes	No
7. Is your digestion poor?	Yes	No
8. Do you have trouble thinking clearly?	Yes	No
9. Do you feel unhappy?	Yes	No
10. Do you cry more than usual?	Yes	No
11. Do you find it difficult to enjoy your daily activities?	Yes	No
12. Do you find it difficult to make decisions?	Yes	No
13. Is your daily work suffering?	Yes	No
14. Are you unable to play a useful part in life?	Yes	No
15. Have you lost interest in things?	Yes	No
16. ¿Se siente aburrido/a?	Yes	No
17. Do you feel tired all the time?	Yes	No
18. Has the thought of ending your life been on your mind?	Yes	No
a. Have you decided how you are going to do it?*	Yes	No
b. Have you decided when you are going to do it?*	Yes	No
19. Do you feel someone has tried to hurt you in some way?*	Yes	No
20. Do you feel that you can do things that others cannot, or that you are a particularly important person?*	Yes	No
21. Have you noted interferences or something weird in your thoughts?*	Yes	No
22. Do you hear voices without knowing where they come from or that other people cannot hear?*	Yes	No
23. Have you had shakings, attacks or falls on the floor with movement of arms and legs, biting of your tongue or loss of consciousness?*	Yes	No
24. Do your family, friends, doctor or priest think that you drink too much?*	Yes	No
25. Have you wanted to quit drinking but you could not do it?*	Yes	No
26. Have you ever had difficulties in your work or studies because of your drinking, for example, due to drinking in your work or center of studies or for not attending?*	Yes	No
27. Have you been in fights or have you been arrested while being drunk?*	Yes	No
28. Have you thought that you drink too much?*	Yes	No

*Translations made by the research team
