# Supplementary material for: Integration of a Technology-Based Mental Health Screening Program Into Routine Practices of Primary Health Care Services in Peru (The Allillanchu Project): Development and Implementation
Source: J Med Internet Res. 2018 Mar 15;20(3):e100. doi: 10.2196/jmir.9208 (PMC5893885; doi:10.2196/jmir.9208)
Supplement: Multimedia Appendix 2 [file jmir_v20i3e100_app2.pdf]

Multimedia Appendix 2

Follow-up interview guide with patients

<table>
<tr><td colspan="4">FOLLOW-UP INTERVIEW GUIDE</td></tr>
<tr><td colspan="2">Patient’s name:</td><td colspan="2"></td></tr>
<tr><td colspan="2">Telephone number:</td><td colspan="2"></td></tr>
<tr><td colspan="2">Phone number:</td><td colspan="2"></td></tr>
<tr><td colspan="2">Health center:</td><td colspan="2"></td></tr>
<tr><td colspan="2">Health service:</td><td colspan="2"></td></tr>
<tr><td colspan="2">PHCP who screened him/her:</td><td colspan="2"></td></tr>
<tr><td colspan="2">Screening date:</td><td colspan="2"></td></tr>
<tr><td colspan="2">Screening result:</td><td colspan="2"></td></tr>
<tr><td colspan="2">PHCP recommendation:</td><td colspan="2"></td></tr>
<tr><td colspan="2">Consented SMS:</td><td colspan="2">Consented audio-recording:</td></tr>
<tr><td>1. YES</td><td>2. NO</td><td>1. YES</td><td>2. NO</td></tr>
<tr><td rowspan="2" colspan="2">Interview modality</td><td>1. Face to face</td><td>Interview date</td></tr>
<tr><td>2. By pone</td><td></td></tr>
<tr><td colspan="4">INTERVIEW GUIDE</td></tr>
<tr><td colspan="4">Questions of the screening</td></tr>
<tr><td colspan="4">1. Do you remember the questions that the nurse/midwife asked you?
2. What do you think regarding asking patients how do they feel during their health consultations?</td></tr>
<tr><td colspan="4">Screening results</td></tr>
<tr><td colspan="4">3. After the nurse/midwife asked you the questions, what did she say? (Explore how did they tell him/her the screening result)
4. What did she recommended to you?
5. What do you think about the recommendations you received?</td></tr>
<tr><td colspan="4">Set of SMS (only if <u>consented SMS</u>)</td></tr>
<tr><td colspan="4">Remember that as part of the project we would send you SMS to motive you to seek the recommended help by the nurse/midwife
6. We send a total of 6 SMS, one daily, one other day: this is, one day yes, the other no. Did you receive all these SMS?
7. Do you remember how many SMS did you receive in total? (Explore at least an approximate). Then: Did any of these SMS were repeated or was sent several times the same day?
8. What did you like about the SMS? Then: What did you not like about the SMS?</td></tr>
<tr><td colspan="4">Mental health care seeking</td></tr>
<tr><td colspan="4">9. Did you seek care from a general practitioner of psychologist as you were recommended, for</td></tr>
</table>

instance, did you try to arrange an appointment?
**10.** Why did you seek (or not) mental health care? If the participant did not seek care, explore the reasons why
**11.** How much do you consider that the SMS motive you to seek health care (with a general practitioner/psychologist)? Nothing, More or less, A lot
**12.** Where did you seek health care? With which professional?
**13.** Did you have any difficulty when seeking health care (with a general practitioner/psychologist)?
**14.** Have you sought care in any other place? (For example, a relative, friend, church, etc.)
**Access to mental health care**
**15.** Did you receive health care?
**16.** If not, why did you no receive health care? *(Explore reasons)*
**17.** Who provided you care? (General practitioner, psychologist, other_______)
**18.** How was the consultation like? *Then:* Did you have other consultations after that?
**19.** What do you think about the care received? Why?
