# Supplementary material for: Integration of a Technology-Based Mental Health Screening Program Into Routine Practices of Primary Health Care Services in Peru (The Allillanchu Project): Development and Implementation
Source: J Med Internet Res. 2018 Mar 15;20(3):e100. doi: 10.2196/jmir.9208 (PMC5893885; doi:10.2196/jmir.9208)
Supplement: Multimedia Appendix 3 [file jmir_v20i3e100_app3.pdf]

Multimedia Appendix 3

Midterm intervention interview guide with PHCP

MIDTERM INTERVENTION INTERVIEW GUIDE		
**Health center:**		
**Health system:**	1	Ministry of Health (SIS)
	2	Social Security System
**Service:**	1	Prenatal service
	2	Tuberculosis service
	3	HIV/AIDS service
	4	Chronic diseases services
**Shift:**	1	Morning
	2	Afternoon
**Health worker(s):**		
**Date:**	/ /	

Remember: This interview is regarded as a support process to the health worker rather than an evaluation. When possible, try to solve all doubts or problems the health worker may have.

INTERVIEW GUIDE

Screening implementation in the daily routine

1. Regarding the screening, how has these past weeks have been?
2. Was the screening as you expected it to be? (Explore met and unmet expectations).
3. How did you feel asking your patients about their mental health?
4. To date, which have been the main difficulties to conduct the screenings with your patients? How have you managed them?
5. How did your patients react to your initiative of asking them about their mood (mental health)?

Technology

6. Has there been any problems with the Tablet and its use? Which ones?
   - How have you managed them? Were there other problems?

SRQ questionnaire

7. Did your patients understand the screening questions? Was there any particular question that was more difficulty for them? How about the ones of psychosis?
    - What did you do when someone did not understand?
8. How did your patients react to the screening questions?
9. Did you feel comfortable asking the questions or was there any one that made you feel uncomfortable? Why?
10. Were there questions that made you question whether the answer should be considered Yes or No? Which ones?
    - How about the questions related to psychosis?

Results

11. How did you feel delivering the results to your patients?
    - Did you have difficulties? Which ones? How did you manage them?
12. Did you have cases with suicide risk? How did you manage it?
13. Up until now, did you ever feel that one of your patients needed specialized care in mental health but the results of the screening did not indicate it?
    - Conversely, has there any patient with whom you thought did not need help but the screening result was positive?
14. How did your patients react to the screening results? *If they mention any difficult case, ask How did you manage it? Explore in positive cases for psychosis*

Referrals

15. Which is the usual referral point for your patients? Why? Explore all possible referral places.
    - Have you had accompany any of them to a specific service?
16. How did your patients react to the referral?
17. Have you received any comments from the health workers of the service where you referred your patients (physician, psychologist, etc.)?
18. Is there anything else that you would you like to add? Any other difficulty, opinion, etc.

Postintervention interview guide with PHCP

POSTINTERVENTION INTERVIEW GUIDE		
**Health center**		
**Health system:**	1	Ministry of Health (SIS)
	2	Social Security System
**Service:**	1	Prenatal service
	2	Tuberculosis service
	3	HIV/AIDS service
	4	Chronic diseases services
**Shift:**	1	Morning
	2	Afternoon
**Health worker(s):**		
**Interviewer:**		
**Date:**	/ /	

AIMS OF THE INTERVIEW

- Explore the experiences and opinions of the health workers participating in the project regarding the training, screening, delivery of results, referral of patients, and support and supervision from the research team.
- Identify the barriers for the implementation of the different activities and processes of the project, collecting suggestions for improvement.
- Assess the willingness of the participating health workers to continue implementing the screening in their health services.

BASIC GUIDELINES

- Interview one health worker that has not been previously interviewed by the same research team member.
- Conduct individual interviews
- Record the audio of the interview
- Know beforehand the amount of screenings and type of cases screened by the health worker. Think how to use this information during the interview, for example, adapting or adding some questions
- Ideally, listen to the previous interview (midterm interview)
- Remind the interviewee that: The opinions will be confidential and she is encouraged to tell us the positive things as well as the things that did not work The idea is to learn from this experience to improve

INTERVIEW GUIDE

Motivation

1. I would like to go to back to some months ago, when you were invited to participate in the project. What motived you to participate in this project? *Explore*

Training

Before we started with the implementation of the screening, we conducted a two-day training session, do you remember this training?

2. What have been the most useful things of this training, the most valuable learnings for you? *Explore*

3. Having implemented the screening, would you have liked receive more training or make it different? *Explore*

Screening and delivery of results

The premise of this Project was that all the patients that came to the service were screened (in the case of tuberculosis, point out that it was a monthly screening)

4. How did you do with the task? (Were you able to screen all your patients?)

- Why was it not possible to screen all your patients?
- How did you organize your work to conduct the screening?
    - In which days or moments of the consultation did you used to conduct the screening? Why?
- Which patients did you screened? Why?

5. Think about all the different difficulties that you may have experience while conducting the screening. Tell me all that you remember. *Explore*

6. How was your experience using the Tablet? *Explore*

- Was it difficult for you at any moment? Did that change over time? How?

7. How did you deliver the results to your patients? What did you used to tell them?

8. Do you remember any situation that was difficult to handle? Could you tell more about it?

9. What suggestions do you have to improve the screening? *Encourage to provide suggestions*

Referral to specialized care

The app recommended you to refer some of your patients to the general medicine or psychology service

10. How many patients do you remember having referred or invited to seek specialized care?

11. What did you recommended to them? Where did you refer them?

12. How was the referral procedure: did you tell them to go, gave them a referral document, arrange for them to get an appointment, or accompany them to the service? Why did you do it that way?

13. Do you know if your referred patients sought and received care? Explore

- Do you know how it went?
- What do you think about the care they received?

Support and supervision

During these two months of implementation, one of the tasks of the research team was to be available to provide support to the health workers in charge or the screening

14. Which team members were in your health center?

15. What do you think about their work?

- Were they helpful? In which way?

16. Are there things they could have done better or different?

General assessment

Now that the implementation of the screening is over, I would like to invite you to do an assessment of your experience. Remember that all opinions are welcome.

17. How do you feel about having participating in this Project? Why?

18. Do you feel that the screening brought positive things to your work or to your consultations? How? Explore

19. In your opinion, is it important to implement a mental health screening at the primary care level? Why?

20. Would you like the screening to continue as part of your work in the health service? Why?

21. Do you consider it is possible to implement the screening as part of your work routine?

- What are the main barriers or difficulties of the implementation?

22. What suggestions or recommendations would you provide to make the implementation of the screening as a regular procedure in your health service and health center more feasible?

23. Would you like to add something else?
