# Supplementary material for: Integration of a Technology-Based Mental Health Screening Program Into Routine Practices of Primary Health Care Services in Peru (The Allillanchu Project): Development and Implementation
Source: J Med Internet Res. 2018 Mar 15;20(3):e100. doi: 10.2196/jmir.9208 (PMC5893885; doi:10.2196/jmir.9208)
Supplement: Multimedia Appendix 4 [file jmir_v20i3e100_app4.pdf]

Multimedia Appendix 4

Analysis of PHCP's interviews

Topics of PHCP's Midterm Interviews

Topics of analysis	Description
1. Implementation of the screening to date	General opinion about the implementation of the screening until the interview day
2. Expectations towards the screening	What did the PHCP expected about the screening, what they thought it would be
3. Experience asking about mental health	Since PHCP were not used to ask about their patients' mental health, how they felt asking about it.
4. Difficulties with the screening	What were the difficulties they encountered while asking the questions of the screening
5. Patients' receptivity to the questions	How their patients reacted to the screening: were they comfortable, cooperative, uncooperative, etc.
6. Difficulties with the app	Due to the screening being adapted into an app, what were the difficulties of its use?
a. Difficulties with the tablet	Difficulties not related to the app but to the tablet: internet connection, freezing of the system
7. Patients' comprehension and management of the questions	Did patients understand the questions of the screening? How did they react to them?
a. Questions that caused difficulties	Which specific questions were difficult to understand or answer for patients? Or to ask for PHCP?
8. Answers of the patients to the questions	What were the most common answers that their patients provide, or which answers were significant to PHCP
9. Experience asking the questions	How did PHCP feel asking the questions of the screening
10. Difficulties rating the questions	List any possible difficulty in the screening rating
11. Experience delivering the results to their patients	How did PHCP feel delivering the screening results to their patients
a. Difficulties with the delivery of results	List all possible difficulties in the delivery of results
12. Suicide risk cases	Suicide risk cases that PHCP had and how they felt and handled them
13. Suspicion of false negatives	The screening app automatically summed the scores of the patients and showed the result. Were there any case in which the app indicated a negative screening but they felt it should be positive?
a. Suspicion of false positives	Similarly, were there any case in which the app indicated a positive screening but they felt it should be negative?
14. Patients' reactions to the results	How their patients reacted to the screening results: did they agreed, disagreed, got upset, sad, etc.?
15. Usual point of referral	Places where PHCP used to refer their patients to receive mental health care: general medicine, psychology, others?
a. Cases that have been escorted to the service	Were there cases in which PHCP decided to escort their patients to the referral service? Why did they decided to do so?

b. Relationship with the professional of the service of referral	How is the relationship with the professional of the service of referral: i.e. was there a previous collaborative work?
16. Patients’ reaction to the referral	How their patients reacted when the PHCP referred them to mental health related service
17. Commentaries of other health workers	Opinions that PHCP heard from other health workers of the health center regarding the implementation of the screening
18. Something else that you would like to add	Any additional information that the PHCP would like to add before ending the interview
19. Additional information	Any other relevant information that the PHCP provided but that was not related to any of the previous topics explored

Topics of PHCP's Postintervention Interviews

Topics of analysis	Description
**1. Engagement**	
1. Motivation to participate in the Project	Why did the PHCP decided to participate in the project, what motivated her
**2. Training**	
2. What was the most useful things of the training	All PHCP were trained by the research team before the implementation of the screening, what were the most useful things they learned in those sessions
3. Recommendations to improve the training	After having received the training and implemented the screening, what would the PHCP suggest to improve the training
**3. Screening and delivery of results**	
4. Was able to screen all her patients	The intention of the project was that the PHCP introduced the screening in their daily routines, so they would screen all of their patients. Was this possible?
5. Why not?	According to the Web based data collection platform, and the PHCP answers, they were not able to screen all their patients. List and explain the reasons why they were not able to do so
6. How did she organized her work to do the screening? (days or moments of the consultation)	Description of how PHCP introduced the screening into their daily routines: i.e. Was there any specific moment?
7. Whom did she screen?	The screening was supposed to be conducted with all their patients, but it did not happen that way. Did they prioritized certain groups of patients or not?
8. Difficulties to conduct the screening (list, explain how she managed them and changes over time)	All difficulties that they had with the implementation of the screening and explore how did they handled them. Description of any possible change over time: i.e. difficulties that were present only at the beginning of the implementation or those that remained constant until the end
9. Experience using the Tablet	Since PHCP are used to conduct all their activities in paper instead of using technology, how did they feel using a mobile app to screen their patients?
10. How did she used to deliver the results	Explore how did PHCP feel delivering results related to their patients' mental health
11. Any difficult situation to handle	Situations that they perceived as difficult to address, and how did they managed them
12. Suggestions to improve the screening	Suggestions to improve the implementation of the screening in a primary care setting
**4. Referral and specialized care**	
13. Number of referred patients	Based on the web based data collection platform, how many cases were reported as referred to another health service
14. Points of referral	Places where the PHCP referred their patients to receive professional care: i.e. general medicine, psychology
15. Additional recommendations that the health worker provided to her patients	The screening app provided a standard set of recommendations according to the patient's result

	that the PHCP could use to provide the results. Did PHCP provided any additional recommendation? If so, in which cases?
16. Referral procedure	Description of how did PHCP made the referral process
17. Follow up to patients (Yes/No)	Did PHCP followed up on their patients after the referral or not?
18. How were their patients after the screening?	Description of what they knew about their patients after the screening, whether if they did a follow up to their patients or not
19. Opinion about the mental health care received by their patients	If they knew that their patients received care, what did they think about such care
20. Opinion about the support and supervision from the research team (In which way it was helpful?)	The research team's role was to provide support and supervision to the PHCP, what did PHCP thought about it? Did they considered it was helpful, in which way?
21. Suggestions to improve the support and supervision	Suggestions to improve the support and supervision provided by the research team
**5. General assessment**	
22. Experience participating in the project	How they felt participating in the project?
23. Positive things that the screening brought to their work	The screening contributed in a positive way to their work or the care they provided in their health services? How?
24. Opinion about the importance of the screening in primary care	What they think about the implementation of the screening in primary care
25. Willingness to continue with the screening as part of their job	Would the PHCP be willing to incorporate the screening as part of their daily routines, beyond the project, as a routine procedure in their services?
26. Feasibility of implementing the screening in the daily routine	Did the PHCP consider that the screening would be feasible to be implemented in primary care as a routine procedure?
27. Main barriers (list)	List all possible barriers that PHCP identify to implement the screening as a regular procedure in primary care
28. Suggestions (list)	List the PHCP suggestions to implement the screening as a routine procedure at the primary care level
29. Additional commentaries	Any additional information that the PHCP would like to add before ending the interview
**6. Additional information**	Any other relevant information that the PHCP provided but that was not related to any of the previous topics explored

Analysis of patients' interviews

Topics of Patient's Interviews

Topics of analysis	Description
1. Remembers the screening questions (Yes/No)	Did the patients being screened by the PHCP?
2. Positive opinion towards the screening	Patients had positive opinions about the screening. Description of these opinions and why they thought it was positive
3. Received SMS	Indicate if patients remember having received the set of SMS
4. How many SMS	Indicate the number of SMS that the patients remembered having received
5. Number of SMS delivered	According to the web based data collection platform, how many SMS were delivered to the patient
6. Sought health care	Help seeking behavior: Did patients sought health care?
7. Reasons to seek health care	For those patients who did seek health care, why did they seek such care?
8. Reasons to not seek health care	For those patients who did not seek health care, why did they not seek such care?
9. SMS motived them	Indicate if patients considered that the set of SMS motivated them to seek mental health care
10. Reason why last question does not apply	Not all patients are able to answer if the set of SMS motivated them to seek health care. Description of these reasons: i.e. patients attended to a mental health service before the delivery of the set of SMS, so did not need to be motivated by the SMS
11. Received health care	Indicate if patients received mental health care after the screening made by the PHCP
12. Why did not received health care	Description of the reasons why did patients did not received mental health care after having screened positive
13. Professional who provided care	For those patients who did received mental health care, who was the professional who provided such care?
14. Observations	Any other relevant information that the patients provided but that was not related to any of the previous topics explored
